# Auditory Brainstem Response to Paired Click Stimulation as an Indicator of Peripheral Synaptic Health in Noise-Induced Cochlear Synaptopathy

**DOI:** 10.3389/fnins.2020.596670

**Published:** 2021-01-11

**Authors:** Jae-Hun Lee, Min Young Lee, Ji Eun Choi, Jae Yun Jung

**Affiliations:** ^1^Department of Otolaryngology Head and Neck Surgery College of Medicine, Dankook University, Cheonan, South Korea; ^2^Department of Otolaryngology Head and Neck Surgery, Dankook University Hospital, Cheonan, South Korea

**Keywords:** auditory peripheral function, auditory brainstem evoked potentials, noise induced cochlear synaptopathy, paired click paradigm, excitotoxicity

## Abstract

**Introduction:**

A defect in the cochlear afferent synapse between the inner hair cells and spiral ganglion neurons, after noise exposure, without changes in the hearing threshold has been reported. Animal studies on auditory evoked potentials demonstrated changes in the auditory brainstem response (ABR) measurements of peak I amplitude and the loss of synapses, which affect the temporal resolution of complex sounds. Human studies of auditory evoked potential have reported ambiguous results regarding the relationship between peak I amplitude and noise exposure. Paired click stimuli have been used to investigate the temporal processing abilities of humans and animals. In this study, we investigated the utility of measuring auditory evoked potentials in response to paired click stimuli to assess the temporal processing function of ribbon synapses in noise-induced cochlear synaptopathy.

**Materials and Methods:**

Twenty-two Sprague Dawley rats were used in this study, and synaptopathy was induced by narrow-band noise exposure (16 kHz with 1 kHz bandwidth, 105 dB sound pressure level for 2 h). ABRs to tone and paired click stimuli were measured before and 1, 3, 7, and 14 days after noise exposure. For histological analyses, hair cells and ribbon synapses were immunostained and the synapses quantified. The relationships among ABR peak I amplitude, number of synapses, and ABR to paired click stimuli were examined.

**Results:**

Our results showed that ABR thresholds increase 1 day after noise exposure but fully recover to baseline levels after 14 days. Further, we demonstrated test frequency-dependent decreases in peak I amplitude and the number of synapses after noise exposure. These decreases were statistically significant at frequencies of 16 and 32 kHz. However, the ABR recovery threshold to paired click stimuli increased, which represent deterioration in the ability of temporal auditory processing. Our results indicate that the ABR recovery threshold is highly correlated with ABR peak I amplitude after noise exposure. We also established a direct correlation between the ABR recovery threshold and histological findings.

**Conclusion:**

The result from this study suggests that in animal studies, the ABR to paired click stimuli along with peak I amplitude has potential as an assessment tool for hidden hearing loss.

## Introduction

Noise-induced hearing loss (NIHL) is a major occupational hazard, resulting in increased social and financial burdens. The military (Bohnker et al., 2002), construction (Seixas et al., 2005), manufacturing, and mining (Landen et al., 2004) sectors of society are severely affected by NIHL due to particular environmental noise issues associated with their activities. A lack of assessment methods and treatments for NIHL is a long-standing issue in many countries.

Moderate levels of noise exposure leading to temporary threshold shifts can cause permanent damage to the synapses between inner hair cells and auditory nerve fibers (Kujawa and Liberman, 2009). Acoustic trauma has been shown to selectively damage auditory nerve fibers with low spontaneous rates and high thresholds; the significant synaptic loss was found above 4 kHz (Furman et al., 2013; Song et al., 2016). Auditory nerve fiber with low spontaneous rates and high thresholds is one of the essential contributors for sound coding, especially in the presence of background noise (Furman et al., 2013; Shi et al., 2016). Functional changes in noise-induced cochlear synaptopathy (NICS) include decreased peak I amplitude in the compound action potential (CAP) and auditory brainstem responses (ABRs; Kujawa and Liberman, 2009; Furman et al., 2013; Wan et al., 2014; Song et al., 2016) with coding defects (Song et al., 2016).

Many research groups have reported the role of ABR peak I amplitude as a marker of NICS using animal models. In contrast to animals, the usefulness of ABR measurements of wave I amplitude, as a marker of NICS, is ambiguous in humans. Various studies showed reduced wave I amplitude with normal audiograms in patients who experienced noise exposure (Stamper and Johnson, 2015; Bramhall et al., 2017), whereas others found no reduction in peak I amplitude in individuals putatively at higher risk of synaptopathy due to significant noise exposure history (Spankovich et al., 2017; Johannesen et al., 2019). High variability of ABR wave I amplitude across individuals could be one of the reasons for this debate (Lauter and Loomis, 1988). As a result, the need for an alternative measure to accurately assess NICS in the clinic has emerged.

Paired click stimuli have been used to evaluate the temporal resolution of the auditory peripheral system in both humans (Ohashi et al., 2005) and animals (Supin and Popov, 1995; Parham et al., 1998; Wysocki and Ladich, 2002). Auditory evoked potentials are recorded after exposure to a stimulus consisting of two clicks, separated in time by various intervals. The relationship between ABR recovery to paired click stimulation and the inter-click interval (ICI) at relatively short ICIs (e.g., <5 ms) provides information on temporal resolution (Henry et al., 2011). Moreover, the evaluation of temporal resolution using paired click stimuli appears to be more reliable than that using other stimuli (e.g., amplitude-modulated tones), possibly because potential artifacts such as adaptation and neural refractoriness (which can be reduced by short initial stimuli; Henry et al., 2011) are avoided or decreased. Because synaptic damage after noise exposure resulted in the decrement of temporal coding ability (Shi et al., 2016), measuring this ability could be a way to diagnose NICS. By analyzing the changes in waveform within the individual responses from two click stimuli, a difference in ABR wave I amplitude across subjects could be reduced.

In this study, we attempted to establish an animal model of NICS by measuring changes in the ABR to paired clicks before and after noise exposure. To determine if this measurement is predictive of NICS, we validated our results with previously used methods of NICS assessment, namely measurements of peak I amplitude and histological determination of synapse numbers in an animal model.

## Materials and Methods

### Experimental Design

**FIGURE 1:**
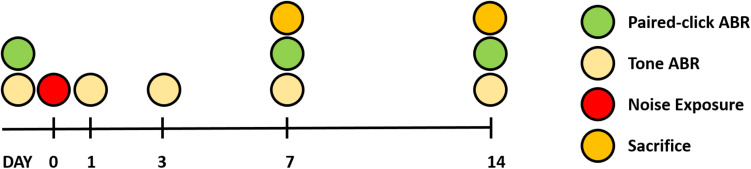
Overall experimental schedule.

Twenty-two male Sprague Dawley rats (6 weeks old) were used for this study. Rats were randomly divided into three groups: baseline (*n* = 6), 7 days after noise exposure (*n* = 8), and 14 days after noise exposure (*n* = 8). All animals received food and water and were maintained under a 12-h light/dark cycle. The ABR to tone burst stimuli was measured before and at 1, 3, 7, and 14 days after noise exposure. ABR recovery was measured before and at 7 and 14 days after noise exposure. At both 7 and 14 days after noise exposure, eight animals in both groups were killed for histological analysis. The comprehensive experimental protocol is schematically presented in Figure 1.

### Noise Exposure

Narrow-band noise at a central frequency of 16 kHz (1 kHz bandwidth) was used for noise exposure. The noise was generated using a type 1027 sine random generator (Bruel and Kjaer, Copenhagen, Denmark) and amplified using an R300 plus amplifier (Inter-M, Seoul, South Korea). Animals were housed in individual cages to prevent protective behavior, and cages were placed in a customized soundproof acryl chamber with the BEYMA CP800Ti (Beyma, Valencia, Spain) loudspeaker attached on top. Animals were exposed to noise at 105 dB sound pressure level (SPL) for 2 h. During the exposure, a frequency-specific sound level meter (type 2250 sound level meter; Bruel and Kjaer, Copenhagen, Denmark) was used to calibrate and monitor the noise level.

### Measurement of Auditory Evoked Potential

#### Measurement of Tone Auditory Brainstem Response

Auditory brainstem responses were measured in all experimental animals. The rats were anesthetized with zolazepam (Zoletil, Virbac, Carros Cedex, France) and xylazine (Rumpun, Bayer, Leverkusen, Germany). Three needle electrodes were placed in each animal. The active electrode was placed at the vertex, and the reference and ground electrodes were placed ventrolaterally to both pinnae. Tone burst stimuli (0.5 ms rise and fall time, alternating polarity) were delivered to elicit ABRs at four test frequencies (8, 12, 16, and 32 kHz). Stimuli were delivered at varying frequencies (intensity decreased from 80 to 10 dB SPL in 5 dB decrements) through the closed field speaker (MF-1, Tucker Davis Technology, Alachua, FL, United States) and were calibrated using a PCB calibrator (480C02, PCB Peizotronics, NY, United States) with a microphone (ER-10B + microphone, Etymotic research, IL, United States). Responses were amplified (10,000×), filtered (0.3–3 kHz band), and averaged. The ABR threshold and peak I amplitude were identified by analysis of stacked waveforms.

#### Measurement of Auditory Brainstem Response to Paired Click Stimuli

**FIGURE 2:**
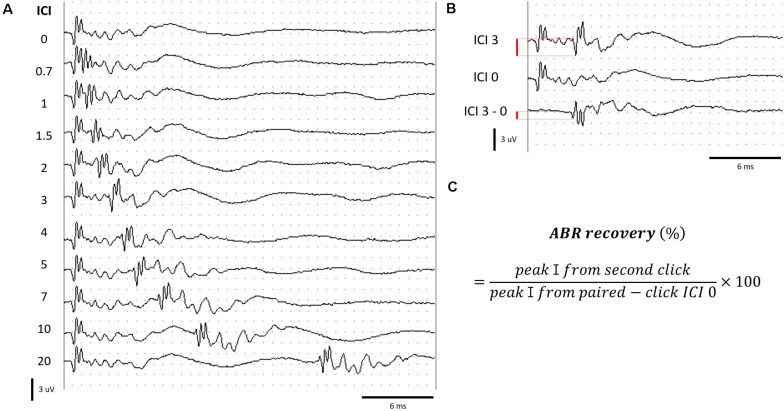
Measurement of ABRs to paired click stimuli. **(A)** ABR to paired click stimuli at various ICIs. **(B)** Determination of peak amplitude by subtracting the waveform of ICI 0 from ICI 3 for an example. **(C)** Formula for calculating the ABR recovery. Red bar **(B)** represents the peak amplitude.

Paired click sounds were generated using SigGen software (Tucker Davis Technology, Alachua, FL, United States). Two click stimuli with 0.1-ms duration constituted a paired click. The first click stimulus was delivered at 0 ms, and the second click was delivered at various ICIs. A total of 10 ICIs, including 20, 10, 7, 5, 4, 3, 2, 1.5, 1, and 0.7 ms, were delivered in decreasing order at 70 peak equivalent SPL. The response was measured using the TDT BioSig III system, using the same electrode placement and response amplification mentioned earlier (Figure 2A).

When stimuli with ICIs shorter than 4 ms were delivered, the response to the second click of the paired click stimulus overlapped with the response evoked by the first click. We isolated the response to the second click stimulus by subtraction of the response of ICI 0 (response from single click; Figure 2B). ABR recovery was calculated using the formula in Figure 2C (Henry et al., 2011).

The ABR recovery threshold was determined as the shortest ICI, which exceeded 50% ABR recovery. The measurement and calculation of the ABR to paired click stimuli have been described by Henry et al. (2011).

### Immunohistochemical Analysis

Immediately after the final ABR measurements, at 7 or 14 days after noise exposure, anesthetized rats were killed, and cochleae were harvested. Small holes were drilled into the apex and oval window of the cochlea, and the cochlea was fixed by perfusion in 4% paraformaldehyde for 2 h and then decalcified for 4–5 days in 0.2-M ethylenediaminetetraacetic acid before microdissecting into five or six pieces. Immunohistochemical analyses of the hair cell marker myosin VIIa and ribbon synapse marker C-terminal binding protein 2 antibody (CtBP2) were performed using a rabbit anti-myosin VIIa antibody (1:200 dilution; Proteus Biosciences) and mouse anti-CtBP2 antibody (1:500 dilution; BD Biosciences), respectively. Secondary antibodies (goat anti-rabbit immunoglobulin G and goat anti-mouse immunoglobulin G1a) coupled to Alexa Flour 488 (green fluorescence; 1:1,000 dilution) and Alexa Flour 568 (red fluorescence, 1:1000 dilution) were used to visualize the hair cell and ribbon synapse markers, respectively.

Images were obtained using a confocal microscope (Flow View 3000, Olympus, Tokyo, Japan) with a 40 × object lens and 2 × digital zoom. *Z*-stacking, at a focal plane interval of 0.5, was used to view the imaging ribbon synapses. The depth of each stack was approximately 40 μm. Ribbon synapses were counted after determining the volume of the three-dimensional rendering of each sample. Synapses in 10–11 inner hair cells were counted and divided by the number of inner hair cells. The assignment of basilar membrane areas to the relevant frequency was performed by generating a cochlear frequency map (Muller, 1991).

### Statistical Analysis

All statistical analyses were performed using the Social Sciences software (ver. 19, SPSS Inc., Chicago, IL, United States). To compare the ABR threshold, ABR peak I amplitude of supra-thresholds (60–80 dB SPL), ABR recovery threshold, and the number of ribbon synapses among the multiple groups, one-way analysis of variance (ANOVA) or Kruskal–Wallis test was conducted depending on the outcome of normality assumption test. If there were significant differences among the multiple groups, *post hoc* analysis with Tukey’s honestly significant difference or Mann–Whitney test was performed to evaluate differences between two different groups (i.e., before noise exposure vs. 7 days after noise exposure and before noise exposure vs. 14 days after noise exposure) using adjusted *p*-value based on Bonferroni correction (i.e., adjusted *p*-value = uncorrected *p*-value × 3 for three groups and adjusted *p*-value = uncorrected *p*-value.

## Results

### Temporary Threshold Shift and Permanent Decrease in Peak I Amplitude With Synaptic Loss After Noise Exposure

**FIGURE 3:**
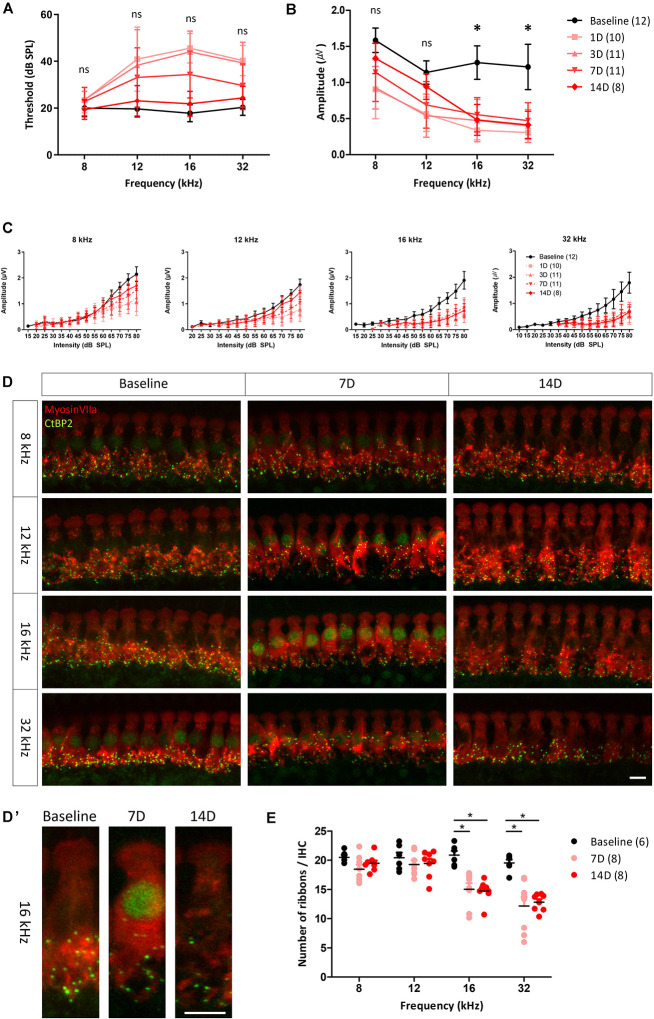
ABR measurement and histological analysis before and after noise exposure. Changes in the hearing threshold **(A)** and averaged peak I amplitude (from 80 to 60 dB in 5 dB decrements); **(B)** from before to after noise exposure. Hearing threshold decreased and then gradually recovered, reaching the baseline level at 14 days after noise exposure. peak I amplitude after stimulation at 8 and 12 kHz fully recovered after 14 days, whereas it remained significantly reduced after stimulation at 16 and 32 kHz up to 14 days after noise exposure. Asterisks (*) mean a significant difference between baseline and 14 days after noise exposure in *post hoc* tests. **(C)** peak I amplitude at all test frequencies decreased after noise exposure. At 8 and 12 kHz, decreased peak I amplitude recovered at 14 days after noise exposure, whereas at 16 and 32 kHz, no significant recovery was apparent. Hair cells (myosin VIIa, red) and ribbon synapses (CtBP2, green) were immunostained **(D,D’)** and analyzed quantitatively **(E)**. **(D)** Days. Significant differences in number of synapses in basilar membrane sections corresponding to frequencies of 16 and 32 kHz, compared with baseline and frequencies of 8 and 12 kHz, were found both at 7 and 14 days after noise exposure. Error bars represent standard deviation. Asterisks (*) mean a significant difference between baseline and 14 days after noise exposure in *post hoc* tests. ns: not significant, Scale bar: 10 μm.

To evaluate changes in the ABR threshold after noise exposure, ABRs to tone burst stimuli were measured several times, before noise exposure and up to 14 days after noise exposure (Figure 3A). Kruskal–Wallis test showed a statistically significant difference in ABR thresholds before and after noise exposure at 12, 16, and 32 kHz [χ^2^(4) = 32.661, *p* < 0.001 for 12 kHz, χ^2^(4) = 50.745, *p* < 0.001 for 16 kHz, and χ^2^(4) = 48.007, *p* < 0.001 for 32 kHz]. *Post hoc* analysis with Mann–Whitney *U* test revealed that ABR threshold at 12, 16, and 32 kHz increased significantly from 1 to 7 days after noise exposure compared with before noise exposure (all adjusted-*p* < 0.001), but no significant difference was observed at 14 days after noise exposure (adjusted-*p* = 1.000 for 12 kHz, adjusted-*p* = 0.350 for 16 kHz, and adjusted-*p* = 0.465 for 32 kHz). 1 day after noise exposure, the ABR threshold showed an approximately 20 dB shift at all test frequencies, except 8 kHz. The threshold shifts were gradually reduced, returning to baseline levels at 14 days after noise exposure. These results demonstrate that the noise stimuli induced a threshold shift, which completely recovered within 14 days after the noise exposure in this rat model.

Functional changes in NICS can be characterized by ABR peak I amplitude decreases (Kujawa and Liberman, 2009; Furman et al., 2013; Lee et al., 2019). To investigate changes in the ribbon synapses of hair cells, the peak I amplitude of ABR to tone burst stimuli was determined and compared at each test frequency. peak I amplitudes at all test frequencies were decreased the day after noise exposure. The decreased peak I amplitudes at 8 and 12 kHz recovered to the baseline level at 14 days after noise exposure, whereas those at 16 and 32 kHz showed no recovery (Figures 3B,C), leading us to speculate that the synapses in the inner hair cells of this model were damaged after noise exposure. To identify the changes of peak I amplitude in the suprathreshold level, we averaged the peak I amplitudes after exposure to suprathreshold stimuli (80 to 60 dB SPL with 5 dB decrements). There was a significant difference in the averaged peak I amplitude at all frequencies before and after noise exposure as determined by the Kruskal–Wallis test [χ^2^(4) = 22.413, *p* < 0.001 for 8 kHz] or one-way ANOVA [*F*(*4, 47*) = 11.728, *p* < 0.001 for 12 kHz, *F*(*4, 48*) = 30.836, *p* < 0.001 for 16 kHz, and *F*(*4, 48*) = 29.989, *p* < 0.001 for 32 kHz]. *Post hoc* analysis revealed that the averaged peak I amplitudes at 8 and 12 kHz recovered to baseline 14 days after noise exposure (adjusted-*p* = 0.06 for 8 kHz, adjusted-*p* = 0.438 for 12 kHz). However, the averaged peak I amplitudes at 16 and 32 kHz were significantly decreased compared with baseline (all adjusted-*p* < 0.001). This result supports previous studies that noise exposure changes peak I amplitude with the suprathreshold level in the ABR measurement (Figure 3B).

To compare the functional and histological changes observed, the cochlea was dissected from the animals and immunostained with a specific antibody targeting a marker of hair cells (myosin VIIa) or ribbon synapses (CtBP2). Stacked images showed that synapses were less abundant in the basilar membrane sections corresponding to the 16 and 32 kHz frequencies at both 7 and 14 days after noise exposure (Figures 3D,D’). There was a statistically significant difference in the number of synapses at 16 and 32 kHz before and after noise exposure as determined by one-way ANOVA [*F*(2,19) = 14.817, *p* < 0.001 for 16 kHz and *F*(2,19) = 15.913, *p* < 0.001 for 32 kHz]. *Post hoc* analyses revealed a significant decrease in the number of synapses at 16 and 32 kHz compared with baseline after 7 or 14 days of noise exposure (all adjested-*p* < 0.001) (Figure 3E). The loss of peak I amplitude accompanied by these histological changes shows that NICS occurred in these animals.

### Delayed Auditory Brainstem Response Recovery Threshold After Noise Exposure

**FIGURE 4:**
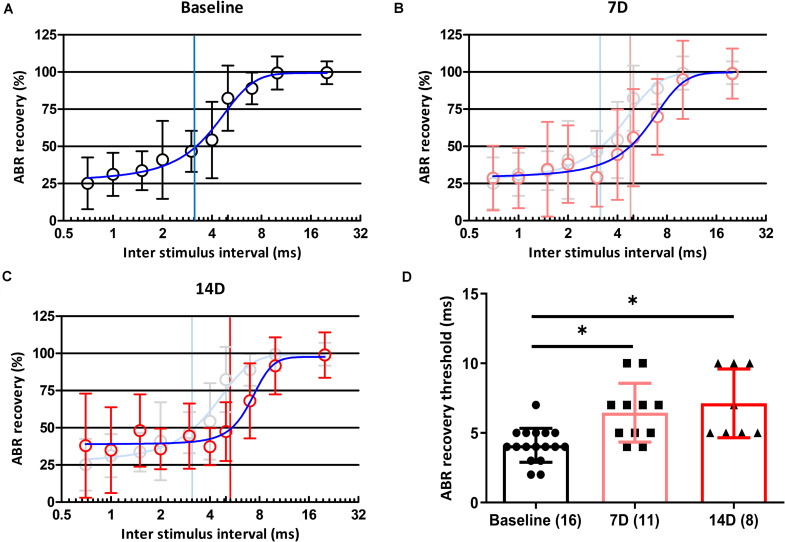
Changes in ABR recovery and ABR recovery threshold before and after noise exposure. ABR recovery was measured after paired click stimulation with 11 ICIs (20, 10, 7, 5, 4, 3, 2, 1.5, 1, 0.7, and 0 ms). Changes in ABR recovery were measured at baseline **(A)** and 7 **(B)** and 14 **(C)** days after noise exposure. ABR recovery thresholds in all subjects were also compared among three time points **(D)**. Numbers of animals used in each time point are shown in brackets. Statistical analyses showed significant differences in ABR recovery threshold values from baseline to 7 and 14 days after noise exposure. There were no differences in these values between 7 and 14 days after noise exposure. Gray plots and error bars represent baseline data. Blue curved lines in **(A–C)** represent non-linear interpolating line between points. Asterisks (^∗^) mean a significant difference between two different groups (i.e., baseline vs. 7D, baseline vs. 14D, and 7D vs. 14D) in *post hoc* tests. Error bars represent the standard deviation.

Of the two types of auditory nerve fibers, those with a low spontaneous rate and high threshold are partially responsible for temporal processing, and selective loss of these fibers can result in coding deficits (Shi et al., 2016). If a synaptic loss in an animal is selective for fibers with a low spontaneous rate and high threshold, the temporal processing of sound could be damaged. We used paired click stimuli to investigate changes in temporal processing ability after noise exposure. At baseline, the ABR recovery threshold was 100% when the ICI was 20 ms and decreased with shorter ICIs. At ICIs of 3 and 4 ms, peak I amplitude in response to the second click was reduced to 50% of that after the first click (Figure 4A). At 7 days after noise exposure, ABR recovery threshold was delayed (Figure 4B), and this delay persisted until 14 days after noise exposure (Figure 4C). We averaged ABR recovery at each ICI in all subjects (Figures 4A–C). ABR recovery thresholds before and both at 7 and 14 days after exposure in all subjects were compared. A Kruskal–Wallis *H* test showed a statistically significant difference in ABR recovery before and after noise exposure, χ^2^(2) = 14.535, *p* = 0.001. *Post hoc* analysis with the Mann–Whitney test showed significant differences between baseline and 7 days after noise exposure (adjusted-*p* = 0.006) and between baseline and 14 days after noise exposure (adjusted-*p* = 0.003; Figure 4D).

### Correlations of the Auditory Brainstem Response Recovery Threshold After Paired Click Stimulation With Peak I Amplitude and Number of Synapses

**FIGURE 5:**
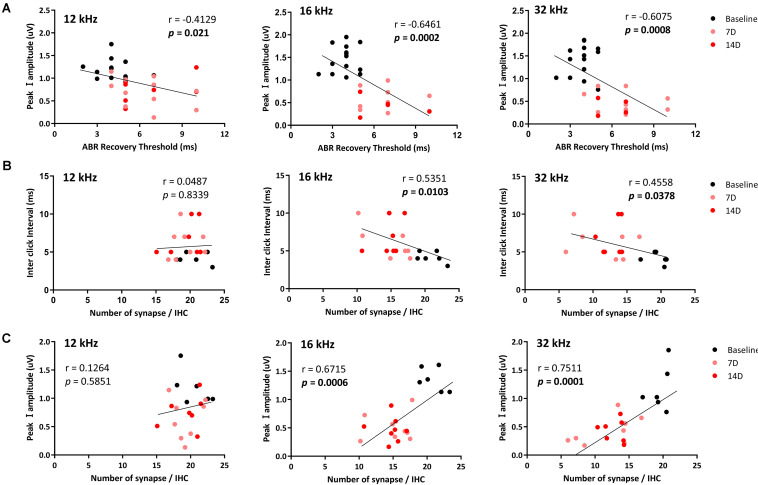
Correlations of the ABR recovery threshold with peak I amplitude and number of ribbon synapses. Correlations of the ABR recovery threshold after paired click stimulation with peak I amplitude **(A)** and a number of ribbon synapses **(B)** were analyzed at 12, 16, and 32 kHz. The correlation between peak I amplitude and a number of ribbon synapse was also analyzed **(C)**. Significant correlations of the ABR recovery threshold with peak I amplitude (at 12, 16, and 32 kHz) and a number of synapses in the basilar membrane (at 16 and 32 kHz) were found (black line represents a linear regression).

To investigate possible correlations of the ABR recovery threshold after exposure to the paired click stimuli with peak I amplitude and number of synapses, these values were compared at three test frequencies (Figure 5) to assess linear correlations. At 8 kHz, there were no changes in peak I amplitude or synapse number; thus, values at this frequency were excluded from the correlation calculations. By comparing ABR recovery threshold with peak I amplitude, there was a significant correlation at all frequencies (12 kHz: *r* = −0.4129, *p* = 0.021; 16 kHz: *r* = −0.6461, *p* = 0.0002; 32 kHz: *r* = −0.6075, *p* = 0.0008; Figure 5A). Furthermore, the ABR recovery threshold was also correlated with the number of synapses at 16 and 32 kHz (16 kHz: *r* = −0.5351, *p* = 0.0103; 32 kHz: *r* = −0.4558, *p* = 0.0378) where peak I amplitude also showed significant correlation with the number of synapses (16 kHz: *r* = 0.6715, *p* = 0.0006, 32 kHz: *r* = 0.7511, *p* = 0.0001; Figures 5B,C and Table 1). These results suggest that the ABR recovery threshold reflects changes in hearing function as much as ABR peak I amplitude and, therefore, can be used to assess changes in hearing function in NICS.

**TABLE 1:** Correlations of the ABR recovery threshold after paired click stimulation with peak I amplitude and number of ribbon synapses.

Correlation	Test frequencies (kHz)					
	12		16		32	
	*r*	*P*	*r*	*p*	*r*	*p*
peak I amplitude vs. ABR recover threshold	–0.4129	**0.021**	–0.6461	**0.0002**	–0.6075	**0.0008**
ABR recovery threshold vs. Number of synapse	0.0487	0.8339	–0.5351	**0.0103**	–0.4558	**0.0378**
peak I amplitude vs. Number of synapse	0.1264	0.5851	0.6715	**0.0006**	0.7511	**0.0001**

*The bold values represent statistical significance.*

## Discussion

In this study, we used tone and paired click stimuli to measure ABRs after noise exposure. The noise exposure (or acoustic trauma) caused threshold shifts, and these shifts recovered to baseline at 14 days, whereas peak I amplitude at 16 and 32 kHz did not fully recover at 14 days after the noise exposure. The ABR recovery threshold after paired click stimulation was delayed at 7 and even 14 days after noise exposure. To investigate histological changes, the number of ribbon synapses was quantified in the basilar membrane regions corresponding to the frequencies at which the ABR was measured. Loss of ribbon synapses was found at 16 and 32 kHz, the frequencies at which peak I amplitude did not recover. Direct correlations of the ABR recovery threshold after paired click stimulation with peak I amplitude and number of ribbon synapses were established.

### Functional and Histological Changes After Noise Exposure

The ribbon synapses between hair cells and auditory nerve fibers in the organ of Corti are reportedly vulnerable to external stress. Numerous studies have assessed functional changes after temporary threshold shifts. In most of those studies, changes in ABR peak I amplitude were measured and compared with histological data, with the conclusion that ABR peak I amplitude is a reliable indicator of synaptic damage after noise exposure (Kujawa and Liberman, 2009; Furman et al., 2013; Shaheen et al., 2015; Suzuki et al., 2016). The changes in synapse numbers and ABR peak I amplitude in our study are consistent with those in previous studies. Furthermore, we found a significant correlation between changes in ABR peak I amplitude (12 kHz: *p* = 0.021, 16 kHz: *p* = 0.0002, 32 kHz: *p* = 0.0008) and ribbon synapse loss (16 kHz: *p* = 0.0103, 32 kHz: *p* = 0.0378) in regions of the basilar membrane.

Auditory nerve fibers in the inner hair cells can be classified according to their spontaneous firing rate and threshold and respond to noise exposure differently (Liberman, 1978). Although we did not address the characteristics of each auditory nerve fiber in this model, a previous study found that auditory nerve fiber with a low spontaneous rate and high threshold can be selectively damaged by noise exposure (Furman et al., 2013).

### Value of Auditory Brainstem Responses to Paired Click Stimuli in Assessing Temporal Processing in Animal Models

Paired click stimuli have been used to measure auditory temporal processing. Several human and animal studies of ABRs to paired click stimuli have been published (Henry et al., 2011; Bidelman and Khaja, 2014). Paired click stimulation has also been used for CAP measurements to assess NICS in the guinea pig (Shi et al., 2016). Previous studies measured CAP using paired click stimuli and shown a similar changing pattern with our ABR recovery threshold result after noise exposure. In this study, the ABR to paired click stimuli before noise exposure was consistent with previous publications (Henry et al., 2011; Bidelman and Khaja, 2014). Changes in ABR recovery threshold after noise exposure can represent the dynamics of synaptic changes by correlating peak I amplitude with histological analysis. Although peak I amplitude has frequency specificity and showed a significant correlation with histological analysis, it cannot explain the synaptic function, such as temporal processing ability. Thus, our results suggest that ABR recovery threshold using paired click stimulation, together with peak I amplitude, has potential for assessing the status of synapse and its temporal processing ability in NICS.

### Clinical Aspect of Paired Click Auditory Brainstem Response

In contrast with animal studies, wave I amplitude in ABR measurement showed inconsistent results among various clinical studies. Diversity in ABR measurement parameters, including head size, sex, quality of electrode contact, or audiometric threshold, has been considered as causes of discordance among studies (Spankovich et al., 2017; Johannesen et al., 2019). High variability of wave I amplitude within subjects is one of the key factors for debate as well (Jerger and Hall, 1980). ABR recovery threshold is measured in a subject by calculating the difference of two responses from paired click stimuli. In this regard, it could increase the consistency of ABR measurement by minimizing individual differences due to ABR measurement parameters.

## Limitations

After noise exposure, there is still no consensus regarding the loss of spontaneous firing rates of the auditory nerve fibers and their effect on temporal processing. A study in gerbils by Schmiedt et al. (1996) concluded that low spontaneous rate fibers are more susceptible to noise damage. However, Bourien et al. (2014) observed otherwise, highlighting the longer and more broadly distributed first spike latency of low spontaneous rate units compared with the other fiber groups. On the other hand, studies on temporal coding concurred that the modulated firing rate of high spontaneous rate fibers is drastically attenuated at moderate to high stimulus levels and low-modulation depths (Joris and Yin, 1992).

Additional experimentation is also needed. It is difficult to confirm the utility of our methodology as a functional assessment tool with such limited animal data. Comparisons between our methodologies and other functional assessments of synaptopathy are required to demonstrate the reliability of our method. Hickox and Liberman (2014) proposed the gap pre-pulse paradigm as a possible method to track functional changes after noise exposure (Hickox and Liberman, 2014). CAP measurements after paired click stimulation should also be assessed to determine the value of ABRs to paired click stimuli in assessing synaptopathy.

## Conclusion

We showed that noise exposure could generate temporary threshold shifts and permanent damage to ribbon synapses. The result of the ABR recovery threshold using paired click stimuli is correlated not only with peak I amplitude but also with the histological assessment. ABR recovery threshold can identify temporal processing of auditory signals. Thus, measurement of ABRs to paired click stimuli is a potentially useful tool in conjunction with ABR peak I amplitude for diagnosing synaptic health in NICS. Furthermore, the individual difference of ABR wave I amplitude can also be reduced by using the paired click paradigm in the clinic.

## Data Availability Statement

The original contributions presented in the study are included in the article/supplementary material, further inquiries can be directed to the corresponding author/s.

## Ethics Statement

The animal study was reviewed and approved by the Institutional Animal Care and Use Committee of the Dankook University (DKU-18-003).

## Author Contributions

J-HL and JJ: conception and design of the study. J-HL and ML: acquisition of data. J-HL, ML, JC, and JJ: analysis and interpretation of data. J-HL, ML, JC, and JJ: wrote the manuscript. All authors contributed to the article and approved the submitted version.

## Conflict of Interest

The authors declare that the research was conducted in the absence of any commercial or financial relationships that could be construed as a potential conflict of interest.
